# Properties and Reaction Mechanism of Brucite-Based Magnesium Phosphate Cement Modified by Ammonium Chloride

**DOI:** 10.3390/ma18133021

**Published:** 2025-06-26

**Authors:** Yueping Chen, Daxing Zhou, Xiaolong Liu, Bin Yang, Hui Lin, Yue Li, Jiale Shen

**Affiliations:** 1 China Railway Construction Group Co., Ltd., Beijing 100040, China; 18606997368@163.com (Y.C.); leo_0729@126.com (D.Z.); 2 Key Laboratory of Urban Security and Disaster Engineering of Ministry of Education, Beijing Key Laboratory of Earthquake Engineering and Structural Retrofit, Beijing University of Technology, Beijing 100124, China; liuxiaolong341623@163.com (X.L.); yangbin186@emails.bjut.edu.cn (B.Y.); shenjl@emails.bjut.edu.cn (J.S.)

**Keywords:** brucite, magnesium phosphate cement, retarder, ammonium chloride, ionic concentration

## Abstract

Aiming at the problem of synergistic regulation of setting time and strength of brucite-based magnesium phosphate cement (BMPC), this study used ammonium chloride (AC) as a variable, and revealed the regulation mechanism of AC on the hydration behavior of BMPC through the tests of setting time, fluidity, and compressive strength, as well as the monitoring of pH-ion concentration, and the microanalysis of XRD-TG-MIP. The results showed that the optimal performance combination of BMPC (setting time of 16 min, fluidity of 120 mm, and compressive strength of 20.5/30.7/54.5 MPa at 3 h/1 d/28 d, respectively) was obtained when AC was doped at a dosage of 4%. The mechanism of retardation stems from the fact that the addition of AC inhibits the dissolution rate of ADP and retards the hydration reaction of Mg^2+^ and $PO_{4}^{3-}$. An appropriate amount of AC can optimize the pore structure of the BMPC matrix and improve the compressive strength of the matrix. The BMPC system based on complete replacement of magnesite by brucite not only significantly reduces carbon emission and cost, but also provides a new path for the development of low-carbon MPC.

## 1. Introduction

Magnesium phosphate cement (MPC) is prepared by mixing magnesium-based raw materials (primarily composed of magnesium oxide), soluble phosphate salts (typically potassium dihydrogen phosphate or ammonium dihydrogen phosphate), and water [1]. This cementitious material demonstrates remarkable properties including rapid setting and hardening, high early-age strength, superior bonding capability, excellent volume stability, and favorable biocompatibility [2,3]. Owing to these advantageous properties, MPC has been widely applied in fields such as rapid repair and reinforcement of buildings [4,5].

Currently, the magnesium raw materials commonly used for preparing magnesium phosphate cement are mainly heavy-burned magnesia, but the main problems are large carbon emissions, etc. Therefore, in order to achieve low-carbon preparation of magnesium phosphate cement, a common method is to use light-burned magnesia or some auxiliary cementitious materials and industrial solid wastes, such as fly ash, slag, lithium slag, etc., to partially replace heavy-burned magnesia. Its replacement amount is generally within 40%, and the carbon emission of the prepared magnesium phosphate cement is still large [6,7]. To this end, some scholars have proposed that the use of heat-activated magnesium-containing minerals or magnesium-containing waste residues to replace heavy-burned magnesia can reduce the carbon emission of MPC, and it can also take into account the development of its mechanical properties. The relevant research results are shown in Table 1.

From Table 1, it can be seen that the complete or partial replacement of heavy-burned magnesia can improve the setting and hardening performance of MPC to a certain extent, but these magnesia raw materials need to be calcined at a certain temperature before they can be used, resulting in a high cost of magnesia raw materials and limited reduction in carbon emissions. Magnesium hydroxide (Mg(OH)_2_) is a common hard mineral [8], which has strong plasticity and good refractoriness, and is widely used in refractory materials, building materials, and metal smelting [9,10,11]. Brucite mineral resources are abundant and inexpensive, and only need to be processed by crushing, grinding, and sieving to obtain magnesium hydroxide powder of different particle sizes [12,13]. (If brucite is used as the magnesium raw material to prepare magnesium phosphate cement, the cost can be reduced while greatly reducing the carbon emission of magnesium phosphate cement. In terms of carbon emissions, the use of brucite is relatively low-carbon, as brucite, which only needs to be mechanically processed, can completely replace magnesium oxide, which is produced by the high-temperature calcination of magnesite followed by a series of mechanical processes).

At present, only Long [14] prepared magnesium phosphate cement from ultrafine brucite (particle size of 325 mesh), and his literature tried various retarders, but none of them could effectively prolong the setting time. After optimizing its mechanical properties, the setting time is less than 10 min. The setting time and mechanical properties of brucite-based magnesium phosphate cement (BMPC) cannot be developed synergistically. In Long’s recent study, tests were conducted using brucite with different particle size gradations to improve the shortcomings of its insufficient mechanical properties by adding metakaolin (MK), but a suitable retarder was still not found [15].

**Table 1:** Influence of the type of magnesian raw material on the setting time and mechanical properties of MPC. ^a^ Quality ratio of other materials substituting magnesian raw materials (in parentheses). ^b^ The ratio of retarder to magnesian raw material.

Types of Raw Materials	Raw Material Calcination Temperature (°C)	M/P ^a^ (Replace MPC)	Retarder Admixture ^b^ (%)		Setting Time/min	Compressive Strength/MPa			References
						3 h	1 d	28 d	
Magnesium slag	1000	2	Borax	0	8			58.1	[16]
				5	10			67.2	
				8	18			56.4	
				10	19			52.7	
				12	21			43.3	
Dead-burned magnesia + Sintered sludge ash (SSA)	800	2.5(0)	None		3		26.8	35.5	[17]
		2.5(5)			4.2		26.9	36.3	
		2.5 (10)			6		27.6	39.1	
		2.5 (15)			9.5		28.4	43.2	
		2.5 (20)			12		19.1	29.3	
		2.5 (25)			13		13.5	25.4	
Dead-burned magnesia/ Steel slag (SS)	>1600/ No need for calcination	3(0)	Borax 5		14.5	38.8	41.9	51.6	[18]
		5			12.5	33.8	39.2	52.1	
		10			10.2	32.6	41.1	54.6	
		15			8.4	27.9	39.6	54.5	
		20			6.5	18.8	38.1	48.9	
Abandoned magnesia refractory brick	No need for calcination	1.5	Borax 5		42	35.1	45.2	65.8	[19]
		2			37	39.1	52.1	68.5	
		2.5			32	26.2	47.3	68.2	
Dead-burned magnesia/ Abandoned lithium waste	1500/ No need for calcination	3 (0)	Borax 5 STP 5		19		43.5	56.1	[20]
		10			21		46.4	62.8	
		20			24		31.8	42.4	
		30			26		33.3	38.3	
		40			30		14.2	18.0	
		50			35		6.5	15.1	
Dolomite +quartz	1250	1.0	Borax 5		25				[21]
		1.5			16	21.9	52.9	54.8	
		2.0			7.5				
Boron mud	800	3	Borax 5		9.5	24.6	35.3	53.9	[22]
	850				11.0	19.3	30.6	48.8	
	900				15.0	11.7	22.5	37.5	

Our team’s preliminary research has discovered that by doping borax and ammonium chloride (AC) at a certain ratio, it is possible to effectively improve the setting and hardening performance of BMPC and take into account the development of strength, which can achieve the preparation of low-carbon high-performance MPC. Therefore, this paper mainly studies the effect of AC on the setting time, fluidity, compressive strength, and hydration performance of BMPC. At the same time, combined with the test results of XRD, TG, and MIP and the test of ion concentration and other chemical methods, the regulatory mechanism of AC on BMPC was explored, which filled the gap of a short setting time in the BMPC field and provided a new idea for solving the problems of the high cost and high carbon emission of MPC.

## 2. Materials and Methods

### 2.1. Raw Materials

**Figure 1:**
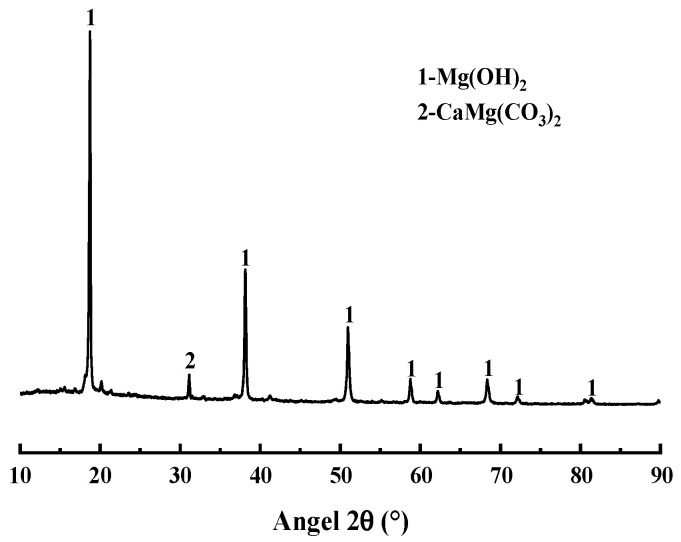
XRD pattern obtained from brucite.

**Figure 2:**
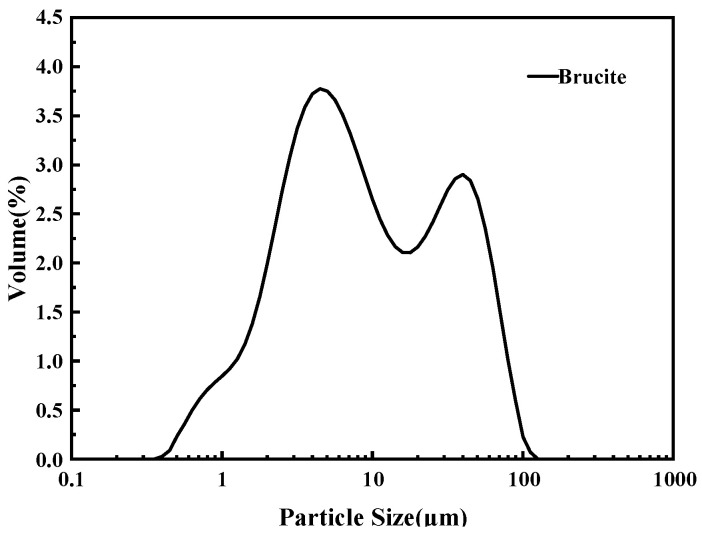
Particle size distribution of brucite.

The magnesia raw material used in the test, magnesite powder (Beijing, China), is mainly made from magnesite ore by crushing and grinding, with an average particle size of 45 μm, and the main chemical composition is Mg(OH)_2_, and its particle size distribution and chemical composition are shown in Figure 1, Figure 2 and Table 2, respectively. The XRD analysis results show that the main mineral components of magnesite powder are Mg(OH)_2_ and CaMg(CO_3_)_2_. The experiments used NH_4_H_2_PO_4_(ADP) (Beijing, China), NaB_4_O_7_·10H_2_O(B) (Beijing, China), and NH_4_Cl(AC) (Beijing, China) are all analytical purity, with a purity of more than 99%.

### 2.2. Sample Preparation

**Figure 3:**
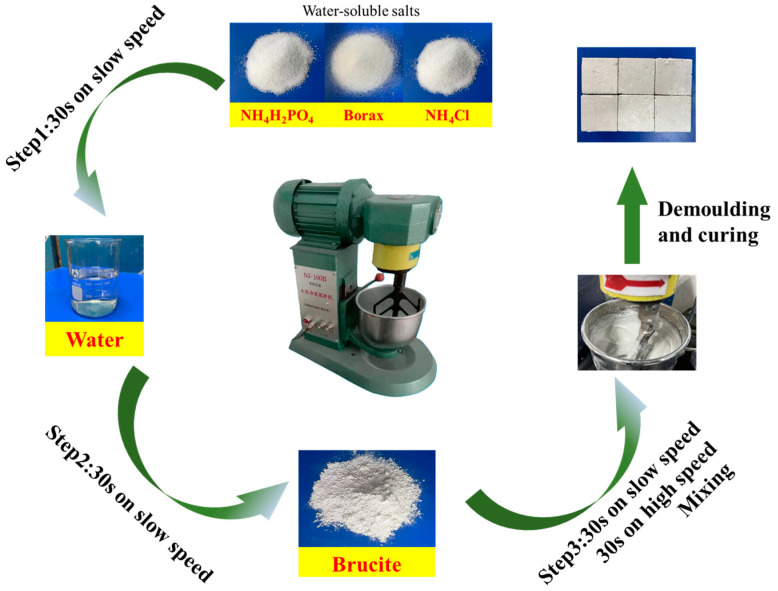
Schematic diagram of preparing BMPC.

The preparation of samples referred to the Chinese group standard T/CMMA 10–2023 [23] (magnesium phosphate composite material). ADP, B, and AC were mixed in a cement paste mixer at a low speed for 30 s according to the ratio in Table 3. After adding, the water-soluble salt was mixed at a low speed for 30 s. After pouring brucite, it was mixed at a low speed for 30 s and then mixed at a high speed for 30 s. Subsequently, the BMPC paste was poured into a 40 mm × 40 mm × 40 mm mold and cured in an environment with a relative humidity of 50 ± 5% and a temperature of 20 ± 2 °C, the specified age for the compressive strength test. The schematic diagram of the preparation of BMPC is shown in Figure 3.

### 2.3. Testing Methods

#### 2.3.1. Macroscopic Performance Testing

The setting time of BMPC was determined according to China National Standard GB/T1346-2011 [24]. For samples with a shorter setting time, the setting time was tested at an interval of 1 min. For samples with a longer setting time, the setting time was tested at an interval of 15 s When the sample was close to final setting, a measurement was made every 30 s. The fluidity of BMPC was measured according to China National Standard GB/T8077-2012 [25]. The fluidity of the paste was determined by measuring the paste diameter from two vertical directions, and the average value of the fluidity of the paste was taken. The compressive strength of BMPC was measured according to Chinese Group Standard T/CMMA 10-2023 for 3 h, 1 d, and 28 d compressive strength. The results were the average strength value of six samples per group.

#### 2.3.2. Liquid Phase pH Test and Ion Concentration

Use a magnetic stirrer to mix the solid powder and deionized water in a mass ratio of 1000:1 according to the formula in Table 3 to prepare the suspension. The pH value of the supernatant was measured and recorded every 1 min by a pH meter (PHSJ-6LINESA). The liquid phase ion concentration test uses Optima8300-type inductively coupled plasma emission spectrometer (ICP) and the 83 basic IC plus ion chromatography analyzer to determine the Mg^2+^ and $PO_{4}^{3-}\;$ in the BMPC suspension, respectively (according to the chromat conditions specified in HJ 669-2013 standard [26], the orthophosphate detected in the form of $PO_{4}^{3-}$ includes $H_{2}PO_{4}^{-},HPO_{4}^{2-}$, and $PO_{4}^{3-}$). The water–cement ratio of BMPC slurry is amplified to 10 to prepare the suspension, and the slurry is stirred with a magnetic stirrer throughout the hydration process of BMPC to ensure that the slurry does notitate. During the test, the suspension is allowed to settle and then the supernatant is taken and filtered with filter paper. Then, 1 mL of sample is sucked with a pipette into a 100 mL conical flask and diluted with deionized water for testing. The processes of standing, filtering, and taking are completed within 5 min.

#### 2.3.3. Isothermal Calorimetry

The exothermic behavior of BMPC during hydration was investigated via isothermal microcalorimetry using a TAM III instrument (Tianjin Institute of Industrial Biotechnology, Chinese Academy of Sciences, Tianjin, China). Solid reactants and deionized water were homogeneously blended at predetermined ratios (Table 3), followed by immediate transfer into a sealed ampoule. The suspension underwent vigorous agitation for 30 s prior to isothermal measurement at 20 °C. Continuous thermal monitoring persisted until signal attenuation below 50 μW, establishing the experimental endpoint based on thermal equilibrium criteria.

#### 2.3.4. Microscopic Testing

Hydrated specimens were extracted at designated intervals and immediately quenched in isopropanol to arrest hydration. The stabilized samples underwent vacuum desiccation at 40 °C until mass equilibrium was achieved. Pulverized materials were sieved through 200-mesh apertures (<75 μm) to ensure homogeneity for subsequent microstructural characterization.

(1) X-ray Diffraction (XRD)

Crystalline phase constituents were analyzed using an X′Pert PRO MPD diffractometer (Malvern Panalytical, London, UK) with monochromatic Cu-Kα radiation (λ = 0.154 nm). Scans spanned 10–90° 2θ with a step width of 0.02° and angular velocity of 2.0° 2θ/min.

(2) Thermogravimetry (TG)

Mass loss profiles were acquired via a TG 209 F1 Libra analyzer (NETZSCH) under nitrogen purge (50 mL/min). Specimens were heated from 30 °C to 500 °C at a ramp rate of 10 °C/min to quantify bound water and decomposition phases.

(3) Mercury Intrusion Porosimetry (MIP)

The pore architecture of BMPC specimens was characterized via mercury intrusion porosimetry (AutoPore IV 9500, Shenzhen, China) under standardized operational parameters. Measurements spanned pore diameters from 5 nm to 400 μm, achieved through controlled intrusion pressures (0.10–60,000 psia) with a mercury contact angle of 130° following ASTM D4404 [27] guidelines. Test samples (1.1 ± 0.2 g) were preconditioned at 60 °C for 24 h prior to analysis to ensure residual moisture removal.

## 3. Results and Discussion

### 3.1. The Effect of AC on Macroscopic Properties of BMPC Paste

**Figure 4:**
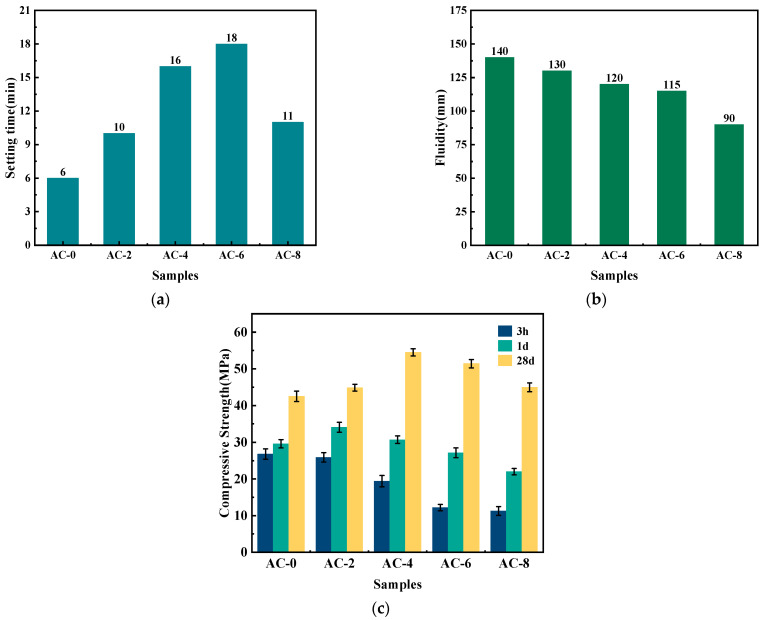
Effect of AC on macroscopic properties of BMPC paste. (**a**) Setting time. (**b**) Fluidity. (**c**) Compressive strength.

As illustrated in Figure 4, the impact of AC on the setting time, fluidity, and compressive strength of BMPC is evident. Figure 4a,b demonstrate that the setting time of BMPC without AC is approximately 6 min. Upon the incorporation of AC, the setting time of BMPC initially increased and subsequently decreased with the rise in AC dosage. Notably, when the AC dosage reached 6%, the setting time of BMPC attained 18 min. However, it is important to note that this increase in setting time was accompanied by a significant decrease in fluidity, with the fluidity of BMPC decreasing by 36% when AC doping was 8% compared to the AC-0 sample. The underlying reasons for these observations will be elucidated in Section 3.4.

The effect of AC on the compressive strength of BMPC is illustrated in Figure 4c. With the increase of AC content, the hourly strength of BMPC showed different degrees of decrease with the increase of AC doping, and its 1 d and 28 d strengths showed the trend of increasing and then decreasing with the increase of AC doping. The AC admixture content was 2% and 4%, and the compressive strength of BMPC at 1 day and 28 days reached the optimum values, respectively. Compared with AC-0 specimen, the 28 d compressive strength of AC-4 specimen increased by 44.6%, whereas a further increase in AC dosing leads to a decrease in the compressive strength of BMPC.

Considering the influence of AC on the setting time flowability and compressive strength of BMPC, it is believed that the optimum admixture of AC is 4%. At this ratio, the setting time, flow, and 3 h, 1 d, and 28 d compressive strength of BMPC are 16 min, 120 mm, and 2.5 Mpa, 30.7 Mpa, and 54.5 Mpa, respectively.

### 3.2. The Effect of AC on Hydration Process of BMPC

**Figure 5:**
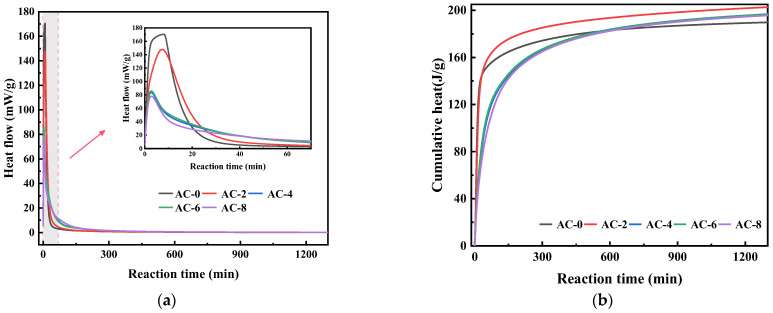
Effect of AC on the hydration of BMPC. (**a**) Hydration exothermic rate of BMPC. (**b**) Cumulative heat of BMPC.

The impact of AC on the hydration performance of BMPC is illustrated in Figure 5. Figure 5a demonstrates that the incorporation of AC altered the reaction process of BMPC, resulting in a gradual decline in the maximum reactive exothermic rate of BMPC when AC doping was less than 2%. However, with the increase of AC doping, the maximum reaction exothermic rate of BMPC gradually decreases. A comparative analysis reveals that the maximum reactive exothermic rate of BMPC decreased by 13.2%, 50.9%, 49.5%, and 56.7%, respectively, with increasing AC doping. As illustrated in Figure 5b, the cumulative exothermic curves of BMPC demonstrate that the cumulative exothermic amount of BMPC increased at 2% AC doping compared with AC-0 specimens. Furthermore, the cumulative exothermic amount of BMPC decreased to different degrees with increasing AC doping. However, the total exothermic amount of the specimens was higher than that of AC-0 when it was stabilized. The results indicate that the addition of AC can reduce the maximum reaction exothermic rate of BMPC and improve its reaction degree. However, when the addition of AC exceeded 4%, its effect on the degree of reaction of BMPC was not negligible. The time of hydration test (approximately 1 day) corresponds to the compressive strength result of 1 day in Section 3.1.

To further elucidate the impact of AC doping on the BMPC reaction process, the reaction kinetic Equation (1) proposed by Knudson was followed. During the initial stage of the reaction, the hydration reactions of all samples were intense, and the cumulative heat release testing time for all samples lasted for over 1200 min. Considering that the cumulative heat release of all samples tended to level off after 800 min, only the first 800 min of hydration heat release simulation calculations were considered in the actual hydration simulation [28]. Was employed to process the heat of hydration data of the aforementioned samples.

(1) $$\frac{1}{Q_{\left(t\right)}}=\frac{t_{50}}{Q_{max}}\cdot \frac{1}{\left(t-t_{0}\right)}+\frac{1}{Q_{max}}$$

where $Q_{\left(t\right)}$ is the amount of heat released at time t. $Q_{max}$ is the maximum amount of heat released during the ∞ reaction. For cementitious mixtures, the maximum hydration is expressed as $Q_{max}$ and is highly correlated with the cement particle size distribution, water–cement ratio, curing temperature, and humidity [29]. *T*_50_ is the reaction time when the heat generated reaches 50% of the total heat release, and (*t* − *t*_0_) is the reaction time starting from the acceleration cycle, *t*_0_, as the starting point.

**Figure 6:**
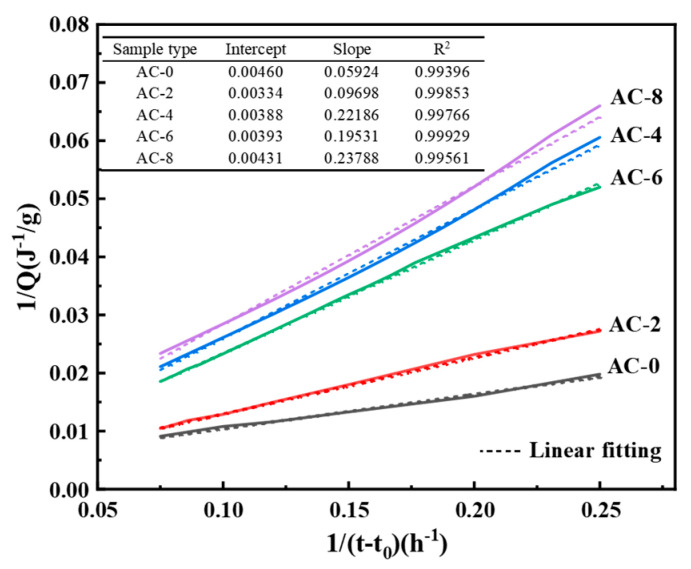
Curve fitted using Knudson’s equation.

Fitting the heat of hydration data shown in Figure 6 yields the Q_max_ values shown in Table 4. The calculated Q_max_ values are shown in Table 4, where Q_(24)_ represents the exothermic heat of hydration reaction for 24 h and Q_(24)_/Q_max_ can roughly represent the degree of hydration reaction for 24 h [30]. It can be seen that the value of Q_max_ first increases and then decreases with the increase of AC doping, while the value of Q_(24)_/Q_max_ first decreases and then increases with the increase of AC doping. This indicates that the addition of a moderate amount of AC decreases the early reaction degree of BMPC, but has little effect on its early reaction degree when the AC doping is greater than 6%. This further suggests that the addition of AC retards its early reaction, which in turn prolongs its setting time. This explains why in Section 3.1 the setting time of BMPC gradually increases with the increase of AC doping when the content of AC is not more than 6%, and decreases when the AC content is 8%.

### 3.3. The Effect of AC on Liquid Phase pH Value and Ion Concentration

**Figure 7:**
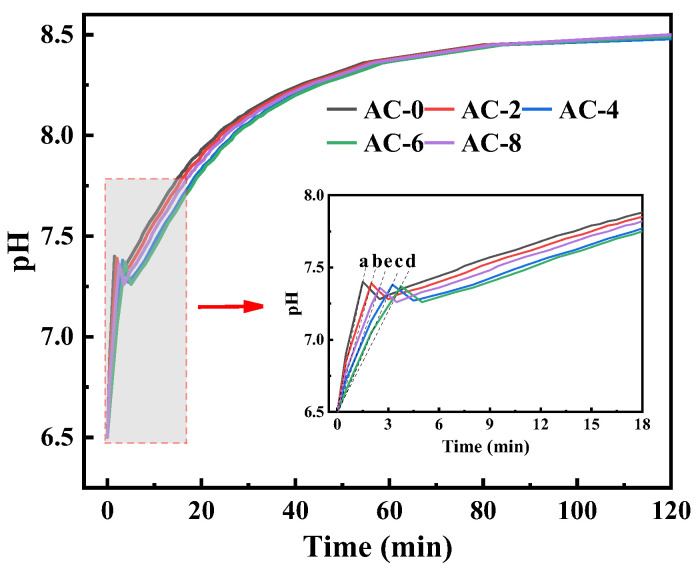
pH and electrical conductivity curves of the BMPC solutions (The slopes of the samples AC-0, AC-2, AC-4, AC-6, and AC-8 were denoted by a, b, c, d, and e, respectively).

The pH changes of the BMPC suspensions are shown in Figure 7. From the figure, it can be seen that, as with the control group AC-0, the pH values of BMPC suspension after the addition of AC are presented first rapidly rise to a higher point, slightly back to a stable state, and tends to stabilize the state of the pH value of the BMPC suspension with the increase in the amount of AC doping, showing a decreasing trend. As can be seen from the enlarged graph in Figure 6, there is an inflection point in the process of the rise of the pH value of the suspension of each specimen, and the time of the inflection point with the increase of AC doping appeared to varying degrees of delay, which may be due to the release of H^+^ in the BMPC suspension in the process of hydration product generation and conversion faster than the rate that it is consumed [31]. Before the inflection point appeared, in order to characterize more intuitively the rate of change of BMPC suspension pH over time in the first rise (from 0 to the higher peak), the slope k was calculated for each curve by taking the point from t = 0 to the appearance of the peak [32], and the slopes of the samples AC-0, AC-2, AC-4, AC-6, and AC-8 were denoted by ka, kb, kc, kd, and ke, respectively. Calculations yielded k_a_ = 0.60, k_b_ = 0.45, k_c_ = 0.27, k_d_ = 0.23, k_e_ = 0.34; it can be concluded that when the AC doping is within 6%, the growth rate of BMPC decreases gradually with the increase of AC doping; when the AC doping is 8%, the growth rate of its pH is greater than that of sample AC-4, and the pH at the inflection point also exhibits the same law. The pH at the inflection point showed the same pattern. After the inflection point, the pH value of each specimen of BMPC suspension showed a short decline, and then the pH value of each specimen gradually increased until it stabilized, which indicates that the addition of AC affects the pH value of the BMPC slurry in the early stage, and then affects the hydration reaction in the early stage of the process, in line with the results of the hydration heat test in Section 3.2.

**Figure 8:**
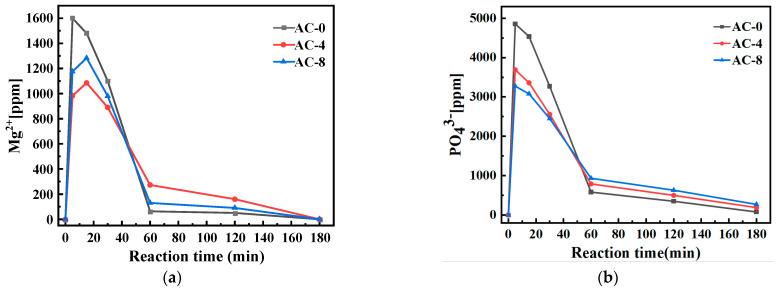
Effect of AC on ion concentration changes in BMPC liquid phase: (**a**) $Mg^{2+}$; (**b**) $PO_{4}^{3-}$.

Figure 8 shows the concentration changes of liquid-phase ions in BMPC suspensions with different AC doping. At 5 min of the reaction, a large amount of $Mg^{2+}$ and $PO_{4}^{3-}$ existed in the solution without AC doping, and this phase of dissolution caused an acidic environment, which led to the dissolution of brucite. The concentration of $Mg^{2+}$ and $PO_{4}^{3-}$ in the BMPC suspension doped with AC was lower than that of the AC-0 specimen at 5 min, which shows that the doping of AC affects the dissolution of NH_4_H_2_PO_4_ and the dissolution of brucite at the initial stage of hydration. As the reaction continued, the concentrations of $Mg^{2+}$ and $PO_{4}^{3-}$ in the BMPC suspension without AC doping decreased rapidly from 5 to 60 min of the hydration reaction, indicating that a hydration reaction took place in the process and a solid-phase precipitation was formed. When AC was added, the concentration of $Mg^{2+}$ in the BMPC suspension increased slowly from 5 to 15 min and decreased slowly from 15 to 30 min. The concentration of $PO_{4}^{3-}$ decreased slowly from 5 to 30 min, and the concentration of $Mg^{2+}$ in the AC-8 specimen was always higher than that in the AC-4 specimen. The opposite was true of $PO_{4}^{3-}$, which was presumed to be the reason for the slow dissolution of magnesite in the BMPC suspension. The dissolution of brucite was slow, and the dissolution of brucite and the precipitation of hydration products proceeded at the same time; AC is a kind of strong acid and weak base salt, which is completely ionized in water to show acidity. When the content of AC was too high, it led to the enhancement of the acidity of the solution, which on the one hand, promoted the dissolution of brucite, and on the other hand, inhibited the ionization of NH_4_H_2_PO_4_. After 30 min, the concentrations of both $Mg^{2+}$ and $PO_{4}^{3-}$ decreased rapidly until they stabilized at 180 min, indicating that solid-phase precipitation of $Mg^{2+}$, $PO_{4}^{3-}$, and other ions involved in the reaction occurred until the reaction was completed. After 60 min, the concentrations of $Mg^{2+}$ and $PO_{4}^{3-}$ increased and then decreased with the increase of AC doping, which indicated that the addition of AC slowed down the solid-phase precipitation reaction in BMPC, and the appropriate amount of AC could slow down the hardening of BMPC, which was in agreement with the variation rule of the condensation time and the results of the pH value test.

Ammonium dihydrogen phosphate (weak electrolyte: weak acid and base salt)

(2) $${NH}_{4}H_{2}{PO}_{4}\to {NH}_{4}^{+}+H_{2}{PO}_{4}^{-}\;\;\;\;\;\;K_{1}=7.52\times 10^{-3}$$

(3) $$H_{2}{PO}_{4}^{-}\to H{PO}_{4}^{2-}+H^{+}\;\;\;\;\;\;K_{2}=6.23\times 10^{-8}$$

(4) $$H{PO}_{4}^{2-}\to {PO}_{4}^{3-}+H^{+}\;\;\;\;\;\;K_{3}=2.21\times 10^{-13}$$

(5) $$Mg(OH{)}_{2}+{2H}^{+}\to Mg^{2+}+2H_{2}O\;$$

Ammonium chloride (strong electrolyte: strong acid and weak base salt)

(6) $${NH}_{4}Cl\to {NH}_{4}^{+}+{Cl}^{-}$$

Combined with the reaction Equations (2)–(6) of BMPC, and synthesizing the test results of pH and ion concentration of BMPC suspension, the reasons for the influence of AC on the hydration performance of BMPC were analyzed as follows:

Since the ionization equilibrium constants ($K_{1},K_{2}{,K}_{3}$) are all fixed values, the following relationship exists: $K_{1}=\frac{c\left(NH_{4}^{+}\right)\cdot c\left(H_{2}PO_{4}^{-}\right)}{c\left(NH_{4}H_{2}PO_{4}\right)};K_{2}=\frac{c\left(HPO_{4}^{2-}\right)\cdot c\left(H^{+}\right)}{c\left(H_{2}PO_{4}^{-}\right)};K_{3}=\frac{c\left(PO_{4}^{3-}\right)\cdot c\left(H^{+}\right)}{c\left(HPO_{4}^{2-}\right)}$. AC is a salt of a strong acid and a weak base is a strong electrolyte, while ammonium dihydrogen phosphate is a salt of a weak acid and a weak base is a weak electrolyte.

Checking the relevant data, it is known [33,34], at 20 °C, the solubility of AC is 37.2 g/100 g of water, and the solubility of NH_4_H_2_PO_4_ is 36.8 g/100 g of water, as the solubility of AC is higher than that of NH_4_H_2_PO_4_ and the ionization ability of AC in water is stronger than that of NH_4_H_2_PO_4_. When the two are dissolved in water together, the ability of AC to bind water is stronger than that of NH_4_H_2_PO_4_, and according to the homoionic effect [35], NH_4_H_2_PO_4_ will be partially dissolved or precipitated out. In other words, the ability of AC to capture water is stronger than that of ADP. The mass ratio of ADP to water in the BMPC slurry used in the test is about 2.2:1. At this time, ADP is already supersaturated, and with the increasing amount of AC doping, there will be more ADP precipitation, which makes the mixed slurry thickened and the fluidity decreases. This explains the phenomenon that the fluidity decreases significantly when the amount of AC doping is too high in Section 3.1.

In Figure 7 the addition of AC causes the reaction of Equation (6) to occur, which leads to the reaction of Equation (2) to proceed in reverse, resulting in a decrease in the concentration of $H_{2}PO_{4}^{-}$ in solution. According to the principle of ionization equilibrium constants in chemical reactions [36], the reaction (3) and (4) proceeds in reverse, and the concentration of $HPO_{4}^{2-}$ and $PO_{4}^{3-}$ decreases accordingly, which also explains why the addition of AC in Figure 7 makes the concentration of $PO_{4}^{3-}$ lower than that of sample AC-0.

This results in the partial precipitation of ammonium dihydrogen phosphate from the solution, which in turn leads to a decrease in the acidity of the solution and a decrease in the rate of dissolution of brucite in solution, which leads to a decrease in the concentration of $Mg^{2+}$ in the solution. When AC is doped in excess, its dissolution in water is more acidic which leads to the continued dissolution of brucite, hence the phenomenon of a higher $Mg^{2+}$ concentration in sample AC-8 than in AC-4. This also confirms the decrease in pH of the solution after the addition of AC samples compared to sample AC-0 in Figure 6. The rate of change in the $Mg^{2+}$ concentration of sample AC-0 was different from that of the sample with the addition of AC from 5 to 15 min, which further suggests that the addition of AC was able to retard the dissolution of brucite and thus the reaction. The $Mg^{2+}$ concentration of the AC-added sample reached a maximum at around 15 min, which is presumed to be the time when the dissolution of brucite may be slower, and the dissolution of brucite and the precipitation of the hydration reaction proceeded simultaneously at around 15 min. After 60 min, the concentration of $Mg^{2+}$ and $PO_{4}^{3-}$ of the samples containing AC was higher than that of AC-0, and the impediment of AC to the dissolution of brucite was even greater, which was the root cause of the delay in the hydration reaction.

In summary, the strong electrolyte AC will completely ionize into $NH_{4}^{+}$ and Cl^-^ in water, while NH_4_H_2_PO_4_ is a weakly acidic salt that will partially dissociate into $NH_{4}^{+}$ and $H_{2}PO_{4}^{-}$ in water. When AC is added, the increased $NH_{4}^{+}$ will compete with the $H_{2}PO_{4}^{-}$ ions in NH_4_H_2_PO_4_, causing the ionic equilibrium of NH_4_H_2_PO_4_ to be upset. A moderate concentration of $NH_{4}^{+}$ will have an inhibitory effect on the dissociation of NH_4_H_2_PO_4_, leading to a slowing down of the decomposition rate of NH_4_H_2_PO_4_, which in turn reduces the overall rate of the reaction and delays the condensation time of the reaction. When the doping of AC is too high, due to the strong electrolyte nature inherent in AC itself, it has a strong ability to seize water in the slurry and ionize before NH_4_H_2_PO_4_. In the case of fixing the water–cement ratio in the test, it makes the hydration involved in the reaction decrease, leading to the reduction of the fluidity of the BMPC slurry and the shortening of the setting time. Therefore, the setting time of BMPC increases and then decreases with the increase of AC doping, which is consistent with the change of setting time in Figure 4a.

### 3.4. Effect of Different AC Content on the Solubility of ADP

Since ADP is a weak acid, weak base salt, and a weak electrolyte, in order to characterize the effect of the addition of AC on the solubility of ADP in the liquid phase, in accordance with the ratios in Table 3, ammonium chloride and borax were added to the pre-configured ADP saturated solution (saturated solution refers to the solute dissolved in the solvent has reached the maximum amount of solubility of the solution) with stirring at a constant speed until the pH value stabilized. Then, the pH value and the change rule of the solution before and after the addition of the soluble salt were tested as to whether the crystals in the solution are precipitated or not.

**Figure 9:**
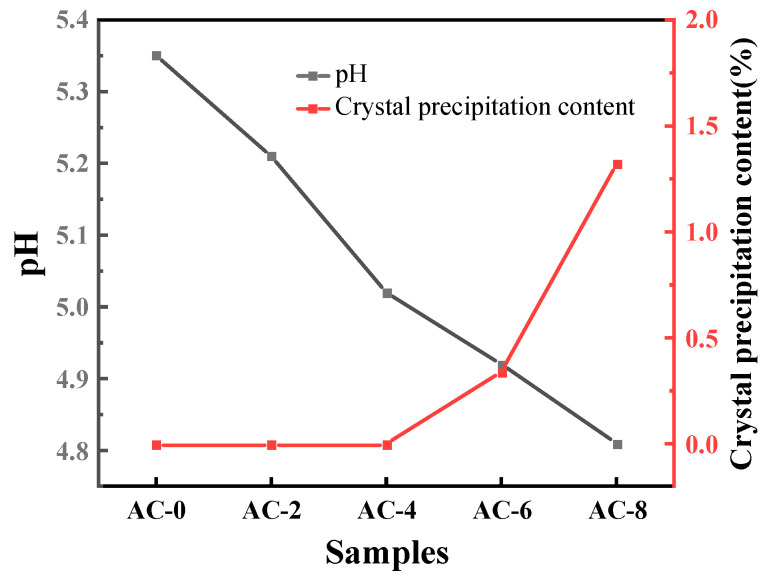
Changes in pH and presence or absence of crystal precipitation of ADP-saturated solution at different AC contents.

The changes in pH and the presence or absence of crystal precipitation of ADP-saturated solution at different AC contents are shown in Figure 9. From the figure, it can be seen that with the increase of AC content, the pH of ADP-saturated solution gradually decreases [33,37], and the content of crystal precipitation in the solution gradually increases. When the AC content is greater than 4%, the phenomenon of crystal precipitation occurs in the solution. The precipitated crystals were dried and sampled for testing, and their composition was ADP. It can be seen that when AC is doped more, the strong electrolyte AC is completely ionized in water, which changes the acidity of the saturated solution of ADP and thus affects the dissolution of ADP. AC competitively occupies a portion of the water, resulting in a decrease in the water that actually participates in the reaction, which explains why the flowability of the BMPC slurry in Section 3.1 decreases progressively with an increase in the amount of AC doped, and the setting time decreases at an AC content of more than 6% decreases.

AC is a strong acid and weak base salt, which is acidic when dissolved in water, and the pH of the solution decreases as more AC is doped. The M/P mass ratio used in the above pH test was 2 (converted to a molar ratio of about 4), and with reference to Equation (7), brucite is in excess in this reaction. At the beginning of the reaction, the basicity of the excess magnesium hydroxide is sufficient to neutralize the acidity of the ADP and there is still a residual OH^−^, which results in a less acidic solution, so that the pH in Figure 7 rises at the initial stage. However, different dosages of AC have an effect on the rate of pH rise in the first rising stage, and the rate of solution pH rise gradually decreases when the dosage of AC is less than 6%. The reason for this, analyzed in conjunction with the experimental phenomena in Figure 8 and Figure 9, is that the complete ionization of $NH_{4}^{+}$ in water due to the strong acidic and weak alkaline salt AC inhibits the first part of the ionization of ADP, causing Equation (2) to proceed to the left. As reaction (5) continues to proceed to the right, the H+ in the suspension decreases. To ensure the dynamic equilibrium of the chemical reaction, this will drive reaction (3) and (4) to the right, which in turn will lead to the continued ionization of ADP in solution in reaction (2). Excess $NH_{4}^{+}$ causes it to undergo the hydrolysis reaction described in Equation (8), where the concentration of H^+^ produced is greater than the concentration of H^+^ consumed by the dissolution of brucite [31,38], hence the transient drop in pH in Figure 6. As the reaction proceeds, $NH_{4}^{+}$, $PO_{4}^{3-}$, and $M_{g}^{2+}$ combine with hydroxide ions in the suspension to produce a struvite precipitate, a phenomenon of ionic change that is verified in Figure 7; this eventually leads to stabilization of the pH of the suspension as the ADP is ionized until it is depleted and the brucite is in excess.

(7) $$NH_{4}H_{2}PO_{4}+Mg{\left(OH\right)}_{2}+{4H}_{2}O\to Mg\left(NH_{4}\right)PO_{4}\cdot 6H_{2}O$$

### 3.5. XRD Analysis

In order to investigate the effect of different doping levels of AC on BMPC, Figure 8 demonstrates the XRD patterns of BMPC specimens with different AC doping levels over time.

**Figure 10:**
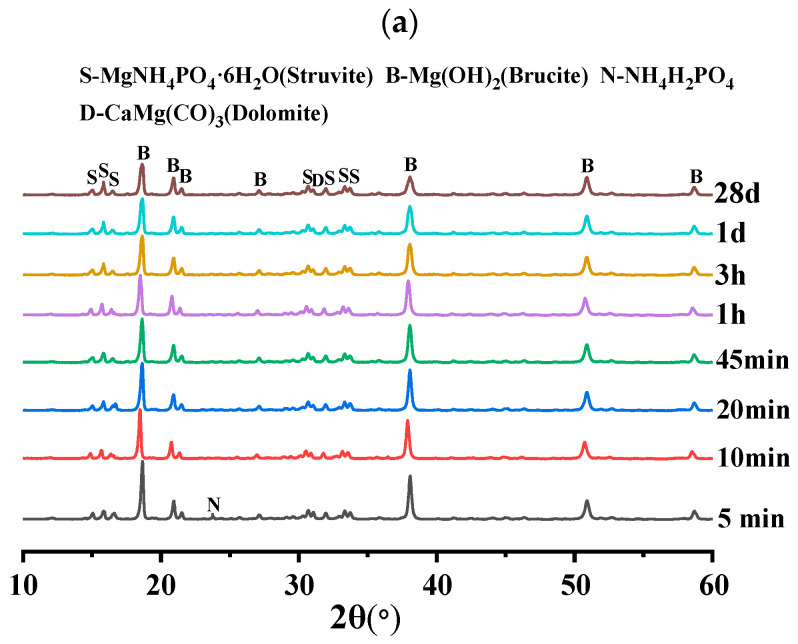

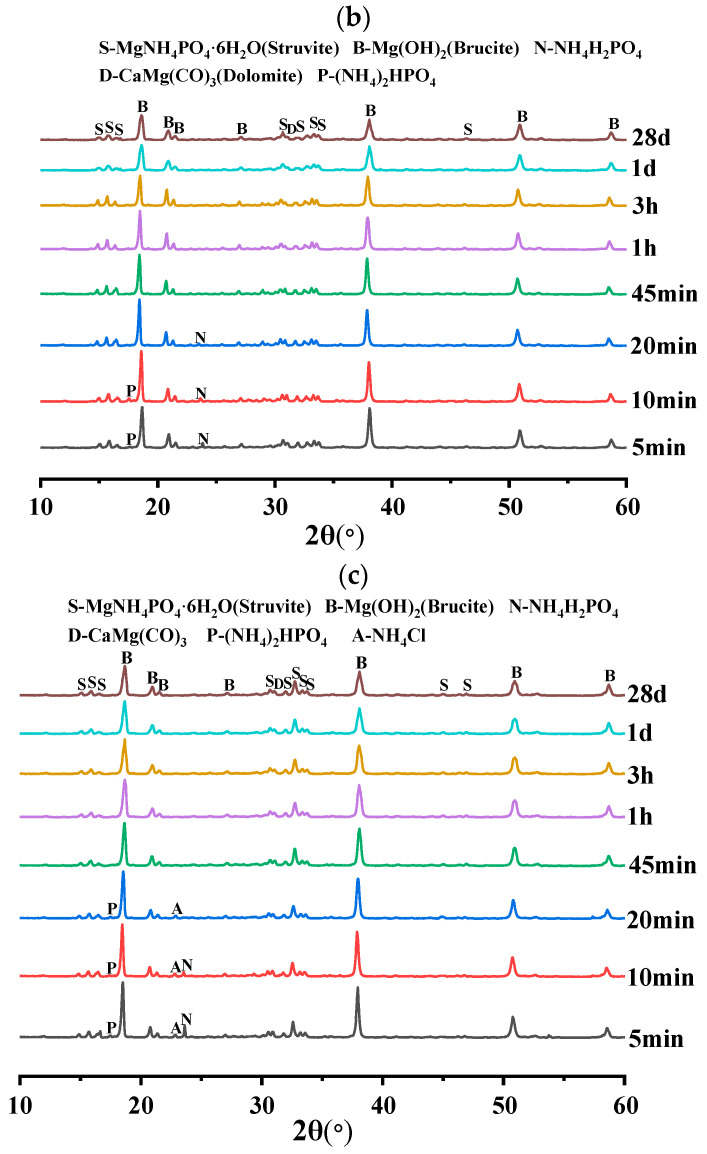
XRD diffractograms of BMPC slurry at different reaction times: (**a**) AC-0; (**b**) AC-4; (**c**) AC-8.

As can be seen from Figure 10a–c, the physical phase compositions of BMPC with different AC doping levels were basically unchanged at 28 d. The hydration product struvite was already detectable from the early 5 min of the reaction, and its production gradually increased with the extension of the reaction time [39], mainly including struvite, brucite, NH_4_H_2_PO_4_, and serpentine (CaMg(CO_3_)_2_), which suggests that the AC addition did not change the final hydration products of BMPC.

As can be seen from Figure 10a, NH_4_H_2_PO_4_ was detected in specimen AC-0 at only 5 min differently, with the increase of AC doping, the retention time of NH_4_H_2_PO_4_ in specimens AC-4 and AC-8 reached 20 min and 10 min, respectively. This suggests that the addition of AC initially inhibited the ionization of NH_4_H_2_PO_4_, reduced the acidity of BMPC slurry, and slowed down the reaction rate between brucite and NH_4_H_2_PO_4_, thus delaying the coagulation time of BMPC, which is consistent with the coagulation time in Figure 4a. The AC-added samples AC-4 and AC-8 specimens detected new (NH_4_)_2_HPO_4_ diffraction peaks near 2θ = 17.47°, and their retention times were 10min and 20min, respectively. This suggests that $NH_{4}^{+}$ ionized by AC with $PO_{4}^{3-}$ and H^+^ in solution produce new substances under certain pH conditions [40], while (NH_4_)_2_HPO_4_ continues to be involved in the reaction with the change of pH in the slurry in the later stages, and therefore it was not detected by the BMPC at 28 d. Unlike the AC-0 and AC-4 specimens, the NH_4_Cl diffraction peak was detected in the first 20 min in the AC-8 specimen and not in the later stages, which suggests that the AC doped with 8% showed an excess in the early hydration reaction of the BMPC slurry. With the change of pH and the reaction temperature in the later stages, the $NH_{4}^{+}$ in the AC took part in the later hydration reaction to produce struvite. This also explains the increase in compressive strength at 28 days of the BMPC specimens doped with AC in Figure 4c compared to the AC-0 group.

### 3.6. TG-DTG Analysis

**Figure 11:**
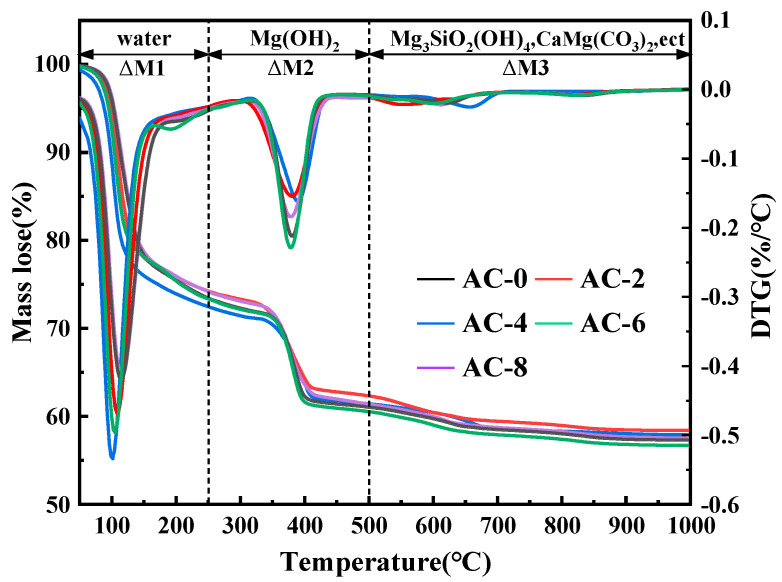
TG-DTG curves of BMPC with varying AC content.

The TG-DTG curves of BMPC at 28 days of age with different AC doping are shown in Figure 11. According to the DTG curves, the weight loss of BMPC specimens was divided into three stages. In the first stage (ΔM1: 30–250 °C), according to the theory proposed by Abdelrazig, this stage is caused by the decomposition of the hydration product struvite (MgNH_4_PO_4_·6H_2_O),the reaction mechanism is as follows in Equation (8) [41,42,43], and the higher the mass loss, the higher the degree of reaction of the BMPC in this temperature range [44]; the second stage (ΔM2: 250–500 °C) is the incompletely reacted brucite (Mg(OH)_2_) [45]; the third stage (ΔM3: 500–1000 °C) is the small amount of Mg_3_Si_2_O_5_(OH)_4_, CaMg(CO_3_)_2_, etc. that are not involved in the reaction [46].

(8) $$MgNH_{4}PO_{4}\cdot 6H_{2}O\left(s\right)\overset{50\sim 200{}^{\circ}C}{\to }MgHPO_{4}\left(s\right)+NH_{3}\left(g\right)↑+6H_{2}O\left(g\right)↑$$

In order to elucidate the effect of AC on the degree of reaction of BMPC, the percentage of mass loss of each sample in the first and second stages was calculated based on the measurement results, as shown in Table 5. It can be seen that in the first stage, the weight loss of BMPC showed an increase and then a decrease with the increase of AC doping, and the maximum weight loss occurred at 4% AC doping. As the AC doping continues to increase, the weight loss of the BMPC samples decreases, but is still higher than that of the AC-0 sample in this temperature range. These phenomena also further confirm that the addition of AC is beneficial to increase the degree of reaction of BMPC, which is consistent with the results of the heat of the hydration test in Section 3.2.

### 3.7. MIP Analysis

The MIP test results of BMPC at 28 d with different AC doping are shown in Figure 11.

**Figure 12:**
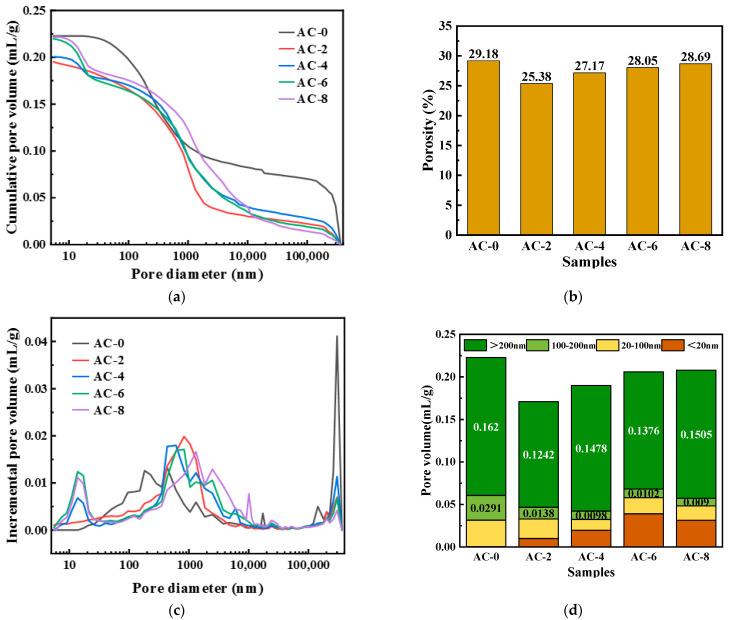
Effect of AC on the pore structure of BMPC. (**a**) Cumulative pore volume. (**b**) Porosity. (**c**) Pore diameter distribution. (**d**) Statistics result of pore volume.

Figure 12a presents the cumulative pore volume of BMPC at different AC doping levels. The cumulative pore volumes of AC-0, AC-2, AC-4, AC-6, and AC-8 were 0.2227 mL/g, 0.1954 mL/g, 0.2005 mL/g, 0.2198 mL/g, and 0.2218 mL/g, respectively. Figure 12b presents the pore volume of BMPC at different porosities of BMPC at different AC doping levels, which are 28.05%, 25.38%, 27.17%, 28.69%, and 29.18%, respectively. From Figure 12a,b, it can be found that the cumulative pore volume and porosity of BMPC gradually increase with the increase of AC doping, but still lower than that of AC-0 specimen. This phenomenon can be explained by the heat of hydration of BMPC under different AC doping, and the AC doping can promote the hydration of BMPC, which leads to an increase in the production of the hydration product struvite, filling the pore space, and thus leading to the cumulative pore volume and porosity of the AC-doped specimen being lower than that of the AC-0 specimen.

The pore size distribution curves of BMPC at different AC doping levels are shown in Figure 12c. It can be observed from the figure that the pore size of the AC-4 specimen is around 500 nm, the pore size of AC-2 and AC-6 is around 800 nm, the pore size of AC-8 is around 1300 nm, and the pore size of AC-0 is around 300 μm. This result indicates that the pore size of BMPC specimens changed significantly at different AC doping levels. The pore size of the unadded AC specimen is much larger than that of the AC-added one, and the maximum available pore size is larger than that of the AC-4 specimen when the AC doping is more than 4% or less than 4%. This indicates that the appropriate amount of AC can promote the BMPC reaction more fully, which also coincides with the fact that the compressive strength of the AC-4 specimens in Figure 4c is significantly higher than that of the other groups.

Based on the effect of pore size on the mechanical properties of cementitious materials, the pore structure can be classified into four categories [44,47]: harmless pores (<20 nm), less hazardous pores (20–100 nm), hazardous pores (100–200 nm), and very hazardous pores (>200 nm). The cumulative volumes of the various types of pores in different ranges are shown in Figure 12d. The volume amounts of the pores that are more hazardous (>100 nm) to the mechanical properties in AC-0, AC-2, AC-4, AC-6, and AC-8 are 0.1911 mL/g, 0.1380 mL/g, 0.1576 mL/g, 0.1595 mL/g, and 0.1478 mL/g, respectively It can be clearly seen that the pore volumes of the AC-added specimens, which are more harmful to the mechanical properties, were reduced by 27.29%, 17.53%, 16.48%, and 22.66%, respectively, compared with those of AC-0. Therefore, the addition of a moderate amount of AC can reduce the harmful pore volume of BMPC, which is favorable to the strength development of BMPC, in accordance with the change rule of compressive strength in Figure 5 [48].

In summary, observing the pore structure of BMPC with different AC doping, it can be concluded that the pore volume of specimen AC-4 is smaller, the degree of reaction is higher, the degree of compactness is higher, and the maximum size of several pores is smaller, and these pore structures determine that it has better mechanical properties.

### 3.8. Reducing Carbon Emissions, Costs, and Energy Consumption in the BMPC Production Process

Brucite ore has been successfully used in the production of BMPC slurries, which proves the possibility of BMPC slurries prepared from the magnesian raw material brucite. The carbon emissions of BMPC were compared with the best overall performance of conventional MPC. Carbon emissions consist of two components: those generated by construction materials during the manufacturing and transportation phases [2]. The carbon emissions generated by the building materials during the manufacturing phase were quantified according to Equation (9), while the carbon emissions during the transportation phase were evaluated using Equation (10).

(9) $$C_{SC}=\sum_{i=1}^{n}M_{i}F_{i}$$

where *C_SC_* refers to the carbon emissions at the production stage of building materials (kg CO_2e_), *M_i_* refers to the consumption of the ith major building material, and *F_i_* refers to the carbon emission factor of the ith major building material. (kg CO_2e_/unit quantity of construction materials).

(10) $$C_{ys}=\sum_{i=1}^{n}M_{i}D_{i}T_{i}$$

where *C_ys_* denotes the carbon emissions from the transportation of building materials (kg CO_2e_), *M_i_* denotes the consumption of the ith major building material (t), *D_i_* denotes the average transportation distance of the ith building material (km), and *T_i_* denotes the carbon emission factor of the ith building material per unit weight of the transportation distance under the mode of transportation [kg CO_2e_/(t·km)].

Re-fired magnesium oxide is produced by calcining magnesite above 1500 °C. According to the literature report [49,50], the carbon emission factor of MgO is 2.23 kg CO_2e_/kg. ADP is about 1.15 kg CO_2e_/kg, borax is 0.91 kg CO_2e_/kg, and ammonium chloride is 0.57 kg CO_2e_/kg. The carbon emission of brucite originates from the indirect CO_2_ emission generated by energy consumed in the process of grinding and sieving [51]. The carbon emission factor of brucite is assumed to be 50.65 kg CO_2e_/t by referring to the carbon emission of cement grinding process [49]. In the calculations, it is assumed that the transportation distance of all the materials is taken as 500 km. Considering the current market size of magnesium phosphate cement (MPC), the mode of transportation is set to be a medium-sized diesel truck (with a load capacity of 8 tons), which has a carbon emission factor of 0.179. Through the calculation, the carbon emission per ton of BMPC is calculated to be 554.23 kg CO_2e_/t, and that of the conventional MPC ranges from 1320.21 to 1834.52 kg CO_2e_/t, with an average of 1593.75 kg CO_2e_/t. This shows that the carbon emissions of BMPC are much lower than the lowest carbon emission level of conventional MPC, reflecting the excellent environmental protection.

In addition, magnesite distribution is limited to certain regions, so the high transportation costs associated with MPC applications should also be considered. Compared to magnesite, brucite minerals are more abundant, widely distributed, and cheaper [6,52]. Currently, brucite powder is widely used in flame-retardant materials, refractory materials, and wastewater and exhaust gas treatment. The successful application of brucite in MPC production provides a brand new idea to solve the problems of the high cost and high carbon emission of MPC.

## 4. Conclusions

In this paper, the effects of different AC dosing on the macroscopic properties such as setting time and compressive strength of BMPC were investigated by using various techniques such as heat of hydration, pH, ion concentration, XRD, TG-DTG, and MIP. Based on the above experimental results, the following conclusions were drawn.

(1) The addition of AC has a significant effect on delaying the setting time as well as 540 improving the compressive strength. Considering the setting time, fluidity, and compressive strength, the optimum dosage of AC was 4%. In this case, the setting time, fluidity, and compressive strength of BMPC at 3 h and 28 d were 16 min, 120 mm, 20.5 Mpa, and 54.5 Mpa, respectively.
(2) The addition of AC, on the one hand, slowed down the dissolution of brucite by inhibiting the dissociation of ammonium dihydrogen phosphate, thus slowing down the overall rate of the reaction; on the other hand, the addition of the appropriate amount of AC supplemented the NH_4_^+^ needed to participate in the reaction to make the reaction more complete, and the generation of the hydration product struvite was increased, which increased the compressive strength of BMPC in the late stage.
(3) The addition of an appropriate amount of AC can optimize the pore structure and reduce the porosity of the BMPC matrix, and the addition of AC can make the reaction more adequate, and have a certain filling effect on the large pores of BMPC, reducing the maximum pore size of BMPC.
(4) The use of brucite as a complete replacement for magnesite not only significantly reduces carbon emissions and costs compared to the use of reburned magnesium oxide for MPC preparation, but also the successful application of brucite in MPC production will broaden the range of raw material choices.

## Figures and Tables

**Table 2:** Composition of brucite powder (%).

Composition	MgO	CaO	SiO_2_	Fe_2_O_3_	Al_2_O_3_	P_2_O_5_	K_2_O	MnO	Others
Mass (%)	81.355	9.168	7.263	1.202	0.569	0.156	0.12	0.091	0.086

**Table 3:** Mix proportion of the samples.

Samples	Brucite/ADP	B/Brucite	AC/Brucite (%)	Water/(Brucite + ADP)
AC-0	2	15	0	0.15
AC-2	2	15	2	0.15
AC-4	2	15	4	0.15
AC-6	2	15	6	0.15
AC-8	2	15	8	0.15

**Table 4:** Q_max_ of BMPC samples with different AC contents.

Sample	Q_(24)_ (J/g)	Q_max_ (J/g)	t_50_ (h)	Q_(24)_/Q_max_
AC-0	191.2	217.4	0.21	87.9%
AC-2	204.9	254.5	0.41	80.5%
AC-4	199.2	257.7	0.95	77.3%
AC-6	199.8	299.4	0.97	66.5%
AC-8	200.1	232.0	0.92	86.3%

**Table 5:** Weight loss rate of BMPC in the temperature range of 50~1000 °C.

Samples	Mass Percentages of Various Components (wt %)	
	ΔM1	ΔM2
AC-0	23.86	12.77
AC-2	24.39	10.99
AC-4	25.07	11.87
AC-6	24.66	13.26
AC-8	24.16	12.32

## Data Availability

The original contributions presented in this study are included in the article. Further inquiries can be directed to the corresponding author.

## References

[1] Wagh A.S., Jeong S.Y. (2003). Chemically bonded phosphate ceramics: I, a dissolution model of formation. J. Am. Ceram. Soc..

[2] Li J., Zhang W., Cao Y. (2014). Laboratory evaluation of magnesium phosphate cement paste and mortar for rapid repair of cement concrete pavement. Constr. Build. Mater..

[3] Chen B., Oderji S.Y., Chandan S., Fan S. (2017). Feasibility of Magnesium Phosphate Cement (MPC) as a repair material for ballastless track slab. Constr. Build. Mater..

[4] Qiao F., Chau C.K., Li Z. (2010). Property evaluation of magnesium phosphate cement mortar as patch repair material. Constr. Build. Mater..

[5] Wang S., Xu C., Yu S., Wu X., Jie Z., Dai H. (2019). Citric acid enhances the physical properties, cytocompatibility and osteogenesis of magnesium calcium phosphate cement. J. Mech. Behav. Biomed. Mater..

[6] Shen X., Wang X., Li K., Hu X., Shi C. (2024). Life cycle assessment of magnesium phosphate cement production. J. Clean. Prod..

[7] Li P., Chen B., Cui Q. (2023). A probabilistic life-cycle assessment of carbon emission from magnesium phosphate cementitious material with uncertainty analysis. J. Clean. Prod..

[8] Fu Y., Yin W., Dong X., Sun C., Yang B., Yao J., Li H., Li C., Kim H. (2021). New insights into the flotation responses of brucite and serpentine for different conditioning times: Surface dissolution behavior. Int. J. Miner. Metall. Mater..

[9] Gong X., Yao J., Yang B., Yin W., Guo J., Song N., Wang Y., Sun H. (2023). Activation–inhibition mechanism of diammonium hydrogen phosphate in flotation separation of brucite and calcite. J. Environ. Chem. Eng..

[10] Gong X., Yao J., Yang B., Yin W., Wang Y., Fu Y. (2023). Selective adsorption of the activator diammonium hydrogen phosphate in the reverse flotation separation of brucite and dolomite. Powder Technol..

[11] Spry P.G., Cody R.D., Cody A.M., Spry P.G. (2002). Observations on brucite formation and the role of brucite in Iowa highway concrete deterioration. Environ. Eng. Geosci..

[12] Simandl G.J., Irvine S.P.M. (2007). Brucite–industrial mineral with a future. Geosci. Can..

[13] Zhang Y., Sun Y., Xu K., Yuan Z., Zhang J., Chen R., Xie H., Cheng R. (2015). Brucite modified epoxy mortar binders: Flame retardancy, thermal and mechanical characterization. Constr. Build. Mater..

[14] Long S. (2023). Study on Preparation and Properties of Low Carbon Phosphate-Activated Cementitious Materials. Master’s Thesis.

[15] Long S., Li Y., Wang N., Wang Z., Lin H. (2024). Research on the influence of ultrafine metakaolin on the properties of magnesium phosphate cement prepared by natural brucite. Constr. Build. Mater..

[16] Dong J., Zheng W., Chang C., Wen J., Xiao X. (2023). Function and effect of borax on magnesium phosphate cement prepared by magnesium slag after salt lake lithium extraction. Constr. Build. Mater..

[17] Zhang J., Qi Y., Yang Y., Long W., Dong B. (2024). Enhancement of magnesium phosphate cement with sintered sludge ash. Dev. Built Environ..

[18] Jing Y., Jiang Y., Chen B., Wang L. (2023). Influence of steel slag powder on the characteristics of magnesium phosphate cement. J. Build. Eng..

[19] Wang X., Li X., Lian L., Jia X., Qian J. (2023). Recycling of waste magnesia refractory brick powder in preparing magnesium phosphate cement mortar: Hydration activity, mechanical properties and long-term performance. Constr. Build. Mater..

[20] Dong P., Ahmad M.R., Chen B., Munir M.J., Kazmi S.M.S. (2021). Preparation and study of magnesium ammonium phosphate cement from waste lithium slag. J. Clean. Prod..

[21] Yu J., Qian J., Wang F., Qin J., Dai X., You C., Jia X. (2020). Study of using dolomite ores as raw materials to produce magnesium phosphate cement. Constr. Build. Mater..

[22] Yu J., Lin L., Qian J., Jia X., Wang F. (2021). Preparation and properties of a low-cost magnesium phosphate cement with the industrial by-products boron muds. Constr. Build. Mater..

[23] (2023). Magnesium Phosphate Composite Material.

[24] (2011). Test Methods for Water Requirement of Normal Consistency, Setting Time and Soundness of the Portland Cement.

[25] (2012). Methods for Testing Uniformity of Concrete Admixture.

[26] (2013). Water Quality-Determination of Phosphate-Lon Chromatography.

[27] (2018). Standard Test Method for Determination of Pore Volume and Pore Volume Distribution of Soil and Rock by Mercury Intrusion Porosimetry.

[28] Knudsen T. On particle size distribution in cement hydration. Proceedings of the 7th International Congress on the Chemistry of Cement.

[29] Taylor H.F.W. (1997). Cement Chemistry, Scotland.

[30] Lin H., Liu H., Li Y., Kong X. (2021). Properties and reaction mechanism of phosphoric acid activated metakaolin geopolymer at varied curing temperatures. Cem. Concr. Res..

[31] Li Y., Wang Q., Sun J., Lin H., Luo X. (2024). Properties and reaction mechanism of magnesium phosphate cement modified by calcium lactate. Constr. Build. Mater..

[32] Caselles L.D., Coumes C.C.D., Antonucci P., Rousselet A., Mesbah A., Montouillout V. (2024). Chemical degradation of magnesium potassium phosphate cement pastes during leaching by demineralized water: Experimental investigation and modeling. Cem. Concr. Res..

[33] Alexandru H.V. (1996). Growth kinetic of prismatic faces of ammonium dihydrogen phosphate crystal in solutions. J. Cryst. Growth.

[34] Ghosh B., Chakraborty J., Abualreish M.J.A., Mondal P., Mahali K., Henaish A.M.A., Roy S. (2024). Study on solubility and solvation thermodynamics for the advancement of biorelevant activities of L-isoleucine and L-serine in aqueous ammonium chloride solutions in the temperature range of 288.15–308.15 K. Biochem. Biophys. Res. Commun..

[35] Peter A., Matthew E., Fabian E. (2020). Principle of Common-ion Effect and its Application in Chemistry: A Review. J. Chem. Lett..

[36] Hongjun Z. (2023). Application and Development of Chemical Concept Based on Subject Understanding: Lonization Equilibrium Constant. Chin. J. Chem. Educ..

[37] Alexandru H.V. (1999). KDP kinetics and dislocation efficiency of growth. J. Cryst. Growth.

[38] Lahalle H., Coumes C.C.D., Mesbah A., Lambertin D., Cannes C., Delpech S., Gauffinet S. (2016). Investigation of magnesium phosphate cement hydration in diluted suspension and its retardation by boric acid. Cem. Concr. Res..

[39] Xu B., Lothenbach B., Leemann A., Winnefeld F. (2018). Reaction mechanism of magnesium potassium phosphate cement with high magnesium-to-phosphate ratiod. Cem. Concr. Res..

[40] Zafarani-Moattar M.T., Gasemi J. (2002). Liquid–liquid equilibria of aqueous two-phase systems containing polyethylene glycol and ammonium dihydrogen phosphate or diammonium hydrogen phosphate. Experiment and correlation. Fluid Phase Equilibria.

[41] Tansel B., Lunn G., Monje O. (2018). Struvite formation and decomposition characteristics for ammonia and phosphorus recovery: A review of magnesium-ammonia-phosphate interactions. Chemosphere.

[42] Sarkar A.K. (1991). Hydration/dehydration characteristics of struvite and dittmarite pertaining to magnesium ammonium phosphate cement systems. J. Mater. Sci..

[43] Abdelrazig B., Sharp J.H. (1988). Phase changes on heating ammonium magnesium phosphate hydrates. Thermochim. Acta.

[44] Li Y., Lin H. (2019). Experimental study on the effect of different dispersed degrees carbon nanotubes on the modification of magnesium phosphate cement. Constr. Build. Mater..

[45] Li Y., Luo X., Lin H., Li H., Liu Y., Mu J., Pan B. (2024). The impact of environmental humidity on the mechanical property and microstructure of magnesium silicate hydrate cement. Constr. Build. Mater..

[46] Yu J., Qian J., Wang F., Li Z., Jia X. (2020). Preparation and properties of a magnesium phosphate cement with dolomite. Cem. Concr. Res..

[47] Lin H., Li Y., Yang B., Xie D. (2024). Effects of different admixtures on the working performance and mechanical properties of phosphogypsum slag-based composite cementitious materials. J. Build. Eng..

[48] Zhang G., Wang Q., Li Y., Zhang M. (2023). Microstructure and micromechanical properties of magnesium phosphate cement. Cem. Concr. Res..

[49] Long S., Li Y., Wang N., Lin H., Wan Z. (2024). Optimization of magnesium phosphate cement prepared by natural brucite using ultrafine metakaolin and metakaolin. J. Build. Eng..

[50] Li Z., Hua Y., Zhang Z., Huang Y., Zhang P., Qian J. (2025). Direct carbonation of magnesium slag after salt lake lithium extraction for use as a cementitious material. Constr. Build. Mater..

[51] Shen W., Cao L., Li Q., Zhang W., Wang G., Li C. (2015). Quantifying CO_2_ emissions from China’s cement industry. Renew. Sustain. Energy Rev..

[52] Wang C., Cheng L., Ying Y., Yang F. (2024). Utilization of all components of waste concrete: Recycled aggregate strengthening, recycled fine powder activity, composite recycled concrete and life cycle assessment. J. Build. Eng..

